# Sociodemographic predictors of parenting stress among mothers in socio-economically deprived settings in rural and urban Kenya and Zambia

**DOI:** 10.1038/s41598-024-63980-2

**Published:** 2024-06-06

**Authors:** Kenneth Odhiambo Okelo, Patricia Kitsao-Wekulo, Silas Onyango, Elizabeth Wambui, Iain Hardie, Josiah King, Aja Louise Murray, Bonnie Auyeung

**Affiliations:** 1 Department of Psychology, School of Philosophy, Psychology and Language Sciences, University of Edinburgh, 7 George Square, Edinburgh, EH8 9JZ UK; 2 African Population and Health Research Center, Nairobi, Kenya

**Keywords:** Parenting, Parenting stress, Childcare, Human behaviour, Public health, Psychology, Health care

## Abstract

Parental stress occurs when parenting demands exceed the resources available to cope with parenting. Previous research has identified household wealth, educational level, marital status, age, and number of dependent children as predictors of parental stress. However, limited evidence exists from sub-Saharan Africa. This study investigated the sociodemographic predictors of parenting stress among mothers in Kenya and Zambia. This cross-sectional study utilised baseline secondary data from parenting intervention programs implemented in Kisumu County (rural Kenya), Nairobi County (Urban Kenya), and Chisamba District (rural Zambia). Out of 913 caregivers recruited for the parenting program, 844 with complete data were included in the analysis. The mean age was 1.0 (SD = 0.7) years. Parental stress was measured using the Parental Stress Score (PSS) tool and demographic questionnaires were used to collect demographic information. Mean PSS were compared across study sites, and a multiple linear regression model was used to examine associations between sociodemographic predictors (household income, educational level, marital status, maternal age, child age, and number of children aged < 5 years) and PSS, adjusting for clustering and other predictors. From the results, the mean PSS in rural Kenya was 37.6 [SD = 11.8], in urban Kenya was 48.4 [SD = 4.2], and in rural Zambia was 43.0 [SD = 9.1]. In addition, the significant association between PSS and mothers’ income and educational level was only observed in Kenyan study sites (income: Kenya rural β = -0.40, *p* < 0.001**; Kenya urban, β = − 0.33, *p* = .02^*^; Zambia rural, β = − 0.01, *p* = 0.7) education: Kenya rural, β = − 0.25, *p* = .005^**^; Kenya urban, β = − 0.14, *p* = 0.07; Zambia rural, β = 0.04, *p* = 0.3). However, marital status, mother’s age, child’s age, and the number of children below five years were not associated with PSS. The results revealed that mothers’ income and education level were negatively associated with PSS, indicating that higher socioeconomic status can buffer the effects of parental stress.

*Trial registration* Pan African Clinical Trials Registry (https://pactr.samrc.ac.za/) database (ID Number: PACTR20180774832663 Date: 26/July/2018; (ID number: PACTR201905787868050 Date: 06/May/2019.

## Introduction

Parental stress occurs when parenting demands exceed the resources available to cope with parenting^1^. This negatively affects the overall functioning of the children and their parents, as stress negatively affects parents’ mental health and well-being, resulting in poor psychological and physical health^2–4^. Parental psychological stress may also affect their children’s well-being, that is, their psychological, health, and developmental outcomes. A distressed parent might not be actively engaged in stimulating interactions with their children, which increases the risk of maltreatment and adverse childhood experiences^5–9^. This makes it important to identify and address predictors of parental stress.

Studies in other countries (Chile, the USA and China) have identified a range of sociodemographic predictors of parental stress. Maternal characteristics such as household wealth, educational level, marital status, age, and the number of children under their care have been indicated as predictors of parental stress^10–13^. Poverty has been associated with poor mental health outcomes^13–15^, and dealing with financial difficulties/hardships leads to high stress levels and negatively affects an individual’s health^16^. Families living in poverty often face challenges such as inadequate financial and educational resources, food insecurity, relationship instability, and overall unpredictability^17,18^.

Lower educational levels have also been shown to contribute to parenting stress^18^. Since parents’ education is associated with their knowledge, attitudes, and practices, educated parents are likely to be informed about better parenting practices and practice positive parenting. They are likely to have access to better income to meet their parenting needs, thereby reducing parenting stress. However, other findings have also demonstrated higher parental stress among mothers with high education (postgraduate degree) and significantly lower parenting stress among mothers with intermediate education (university degree/diploma/vocational training)^11^. These findings could be attributed to the fact that even though education is related to greater resources, which could lead to less parental anxiety, it also facilitates alternative fulfilment, that is, satisfaction with career progression/achievements, which may lead to a greater feeling of dissatisfaction with parenting^19^. In addition, it could also be attributed to ‘parents’ education anxiety’^20^ in which a parent experiences tension, panic, worry, and other forms of negative emotions regarding whether their parenting practices positively affect their children’s developmental outcomes.

Regarding marital status, a study in Kenya and Zambia reported that community support systems lessened the parenting burden on mothers, especially support from their spouses^21^. Specifically, one study found that mothers reported that fathers’ involvement in parenting reduced the stressors that came with parenting^21^. Such findings have also been evident in other contexts, such as a study in the USA on mothers in low-income settings^22^. For mothers in low-income settings, marriage could be a great contributor to an improved household financial base, emotional support, and support in caring for the child(ren)^23^. Marriage quality can also contribute to stress and health outcomes^24^. Marital quality is often defined as the subjective evaluation of relationships and behaviours in relationships, measured by self-reported attitudes towards one’s partner and marriage. This is often rated as behaviour indicating low quality (hostile or withdrawing behaviours) or high quality (supportive behaviours)^24^. Low-quality marriage has been associated with the development of mood disorders and vulnerability to mental health-related illnesses^25^.

Maternal age has also been reported as a predictor of parental stress. Specifically, mothers between 30 and 35 years of age reported less stress than mothers who were younger or older than this age^10^. Younger mothers are likely to experience parenting stress due to limited financial and social support and experience in childcare. On the other hand, older parents might experience stigma towards older parenthood and lack social support from extended families, which might affect their psychological well-being. In a study in the USA by Dougall et al.^26^ mothers older than 40 years reported that even though having children in their forties was advantageous for career establishment and financial security, it also meant that they lacked parenting energy. In addition, the number of children under mothers’ care is also a predictor of parental stress, with mothers with more than one child below eight years reporting higher parental stress levels^12^. Qian et al.^12^ found that two-child families in China had higher parenting stress than only-child families. This could be attributed to the financial and other support needs of more than two children in these age groups. In addition, mothers with more than one child reported requiring more time and energy to meet parenting demands^12^. These effects are significant in understanding contexts such as Kenya and Zambia, where it is estimated that there are an average of four children per family^27,28^. In sub-Saharan Africa (SSA), the geographical area of the African continent that is situated south of the Sahara desert and includes 48 countries and encompassing Middle, Eastern, Southern, and Western Africa^29^) context, qualitative research in Tanzania showed that family finances, marital relationships, child-related factors such as illness, and economic resources within the community contribute to parental stress^30^. Other studies in the SSA context have identified an association between mental health and parental stress, and male caregivers who perceived parenting as a burden had higher parenting stress scores^7,31^. However, there is limited evidence on socio-demographic predictors in the SSA context, where factors such as the poverty index are very high compared to developed countries.

The present study is a secondary analysis of existing studies from SSA. This offers a cost-effective and ethically responsible approach to research, particularly in low-resource settings, where securing funding for primary research can be challenging. Additionally, by utilising secondary data, researchers can maximise the contributions of participants’ data, yielding new scientific insights without the need for additional time, money, or resources. Studies have utilised this approach, especially in SSA, where there is limited research funding, thereby enriching the literature and promoting a more sustainable and ethical model by making use of available data^32^. Therefore, we used datasets from three previous studies conducted in Kenya and Zambia and investigated the association between mothers’ demographic characteristics, such as age, income, education, marital status, number of children below five years of age, and parental stress level. Utilisation of these three distinct studies provides conceptual replication attempts across multiple settings which helps establish how generalizable such findings are. The PSS tool was used to obtain information from parents on their experience of undertaking day-to-day parental responsibility^33^. This study aimed to investigate the association between mothers’ demographic characteristics and PSS.

## Methodology

### Study design

This cross-sectional study utilised baseline secondary data from parenting intervention programs implemented in Kisumu County (rural Kenya), Nairobi County (urban Kenya), and Chisamba District (rural Zambia)^28^. In these three studies, participants were assigned to either the intervention group, which provided a parenting program, or the control group, which received standard care from the Ministry of Health and Education. Participants were drawn from villages/clusters. These clusters were purposively selected to ensure a buffer zone between the intervention and the control arms. In both rural Kenya and rural Zambia study sites, the parenting program intervention was implemented by the Episcopal Relief and Development (ERD) team, together with the Zambia Anglican Council Programmes (ZACOP) in Zambia and with ACK Development Services (ADS) Nyanza in Kenya. This program targeted children below three years of age and focused on enhancing caregivers’ parenting skills to promote their children’s growth and development^34^. For urban Kenya, the African Population and Health Research Centre (APHRC) in partnership with Val Partners and the Ministry of Health implemented an Early Childhood Development (ECD) mobile phone technology application to help young mothers track and respond to their children's developmental progress in a timely manner. This program aimed to improve caregivers’ knowledge, attitudes, and practices on children’s growth and development through training, the use of mobile phone technology to track and stimulate their children’s growth and development, identification of developmental delays, mentorship visits by the Community Health Volunteer (CHVs), and referral to a health facility to address the identified developmental delays.

### Study sites

The study reported in this paper was conducted in both Kenya and Zambia. In Kenya’s rural areas, the study was conducted in the Awasi-Onjiko ward, Nyando sub-county, and Kisumu County. Nyando sub-county had a lower uptake of immunisation, at 76.6%, compared to the county average of 82%. The proportion of mothers who attended four ANC visits was also lower in Nyando (48.4%), compared to the county average of 49.7%). In addition, the greater Nyanza region has a higher HIV prevalence rate of 19.3%, which is above the national average of 5.9%^35^. The study site in which the parenting program intervention was implemented was identified by the Ministry of Health (MOH) Health Information System (HIS) as the most vulnerable area in the entire Nyando sub-county.

In Zambia, the parenting program was implemented in the Mwantaya and Chamuka wards of Chisamba District in Central Province. The Chisamba district has a population of 103,983, with a higher HIV prevalence rate than the national average (13.4%)^36^. Notably, malnutrition rates were high, with stunted growth in children below five years (42.1%). In addition, Mwantaya Ward has only one health clinic despite being sparsely populated^36^.

In urban Kenya, the study was conducted in the Korogocho ward within the Ruaraka sub-county, Nairobi County. Nairobi County, which has 17 sub-counties, has a total population of approximately 4,004,400^37^. Korogocho is a densely populated ward with 41,946 residents and is characterised by high levels of poverty, high birth rates, and a large proportion of young mothers; 25 per cent of mothers of children under five are aged less than 20 years^38^. Poor feeding and childcare practices have been documented among these mothers and are associated with a high stunting rate (50%) and a high infant mortality rate of 57 deaths per 1000 live births^38^. The Korogocho ward was selected for the implementation of the parenting program because of its poor indicators of maternal and child health outcomes^39^.

### Sample size calculation

In rural Kenya, the sample size was calculated based on Hemming and Girling’s study^40^ with six clusters in each arm and a fixed number of clusters. The number of mothers per arm was 132, implying a total sample size of 264 for the two arms. Due to limited resources, with six clusters in each arm, the intracluster correlation (ICC) was set at 0.02, the effect size at 0.43, and the dropout rate at 10%. A similar sampling calculation was performed for rural Zambia^40^, with an assumed minimum detectable effect size of 0.4 with an ICC of = 0.03. The team also estimated a confidence interval of 95%, a power of 80%, and a dropout rate of 10%. Thus, the total sample size for each arm was n = 255 (510 mothers). However, only 395 mothers (children aged < 18 months or pregnant women in the third trimester) met the inclusion criteria. The inclusion criteria included vulnerability status (for example, health and nutrition indicators, HIV exposure and poverty levels) and long-term residency (more than one year) in the study area.

In the Kenyan urban study, the sample size was calculated using the G*Power program, where the program was set to a one-sided t-test involving the difference between two independent means^40^. Using a priori power analysis, we inputted the values of 0.05 and 0.84 the significance levels and a power of respectively. Additionally, equal-sized sample groups were assumed, meaning that the allocation ratio of N1 (intervention group) to N2 (control group) was one. The calculation produced a sample size of 100 for each group, which allowed for a 10% attrition rate gave a result of 110. A total of 254 households were recruited.

### Study sample

**Figure 1:**
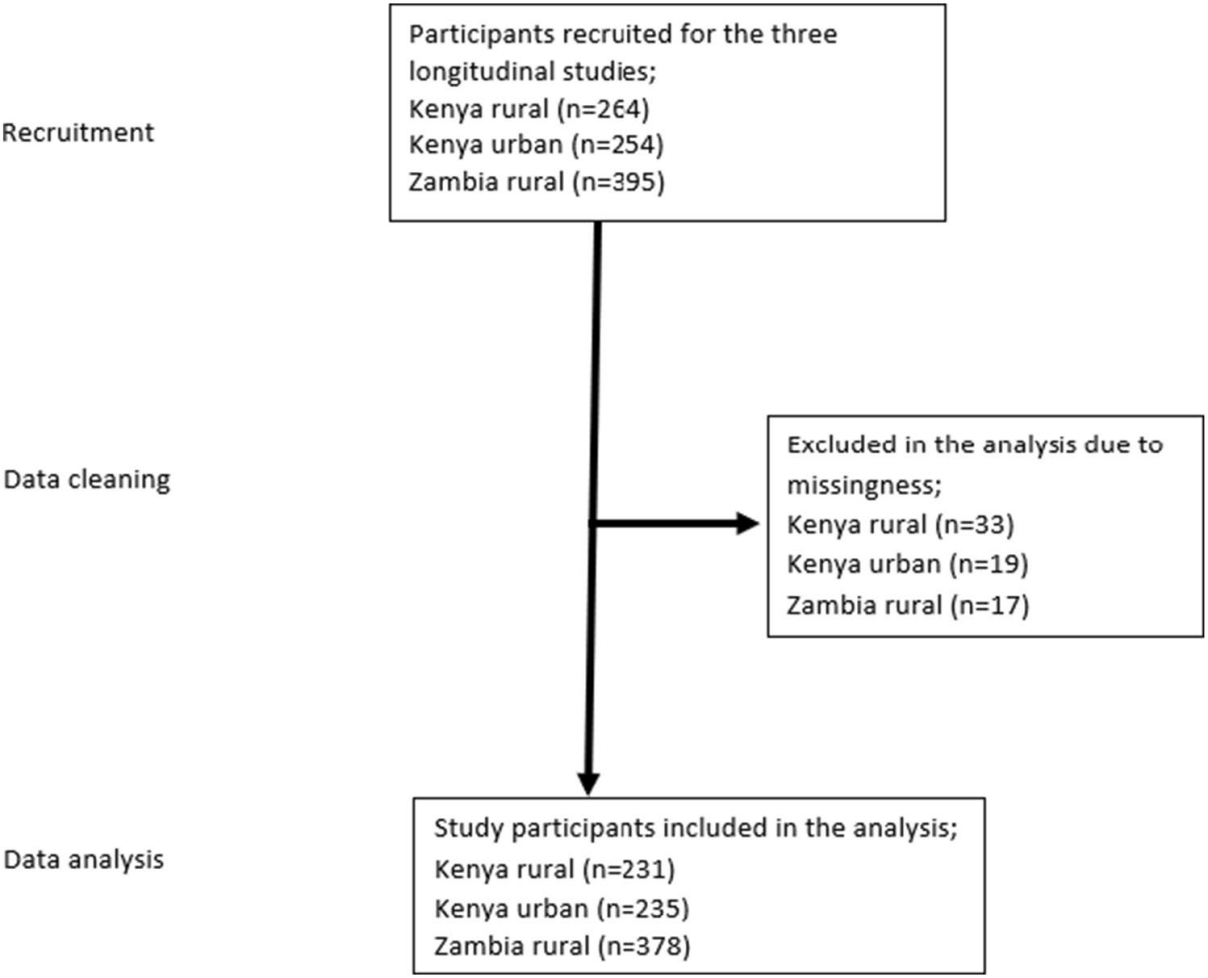
Flow diagram of participants from recruitment to analysis.

Out of 913 caregivers recruited for the parenting program 844 with complete data were included in the analysis. We included mother-infant pairs from the three studies that participated in the baseline (Kenya rural, n = 231; Kenya urban, n = 235; Zambia rural, n = 378) with complete data on the outcome and predictors variables as shown in Fig. 1. Therefore, based on the original studies, assumptions could yield a detectable effect size of 0.4 with an ICC of 0.03, a confidence interval of 95%, and 80% power.

### Measures

Parental Stress Score (PSS) was used for the outcome and sociodemographic predictor information was collected using a questionnaire for mothers questionnaires. Trained Research Assistants administered the questionnaire using an interviewer-assisted method, and the average duration for each respondent was estimated to be one hour. The questionnaire covered items on mothers’ socio-demographics, caregiving knowledge, attitudes, and practices, PSS, health-seeking behaviours, and the Ages and Stages Questionnaire (ASQ).

### PSS outcome measure

This study focused on parental stress levels, measured using the PSS tool to obtain information on parents’ feelings and perceptions of their parenting experience. Studies on the validity and reliability of PSS tools have indicated good internal consistency, construct validity, convergent validity, and test–retest reliability^33,41–45^. Notably, for this study, Cronbach’s alpha coefficient for the internal consistency of the items in the PSS was acceptable for all study sites (Kenya rural: 0.80, Kenya urban: 0.74, and Zambia rural: 0.84). The mothers’ PSS responses were assigned scores based on a *5-point Likert Scale (1* = *Strongly Disagree, 2* = *Disagree, 3* = *Not Sure, 4* = *Agree, 5* = *Strongly Agree*) (Table 1). To compute the PSS, items 1, 2, 5, 6, 7, 8, 17, and 18 were reversed and scored as follows: (1 = 5) (2 = 4) (3 = 3) (4 = 2) (5 = 1) as shown in Table 1. The item scores were then summed. A low score signifies a low level of stress, and a high score indicates a high level of stress. The overall possible scores on the scale ranged from 18 to 90.

**Table 1:** Parental stress score (PSS) tool.

1	I am happy in my role as a parent
2	There is little or nothing I wouldn’t do for my child(ren) if it was necessary
3	Caring for my child(ren) sometimes takes more time and energy than I have to give
4	I sometimes worry whether I am doing enough for my child(ren)
5	I feel close to my child(ren)
6	I enjoy spending time with my child(ren)
7	My child(ren) is an important source of affection for me
8	Having child(ren) gives me a more certain and optimistic view for the future
9	The major source of stress in my life is my child(ren)
10	Having child(ren) leaves little time and flexibility in my life
11	Having child(ren) has been a financial burden
12	It is difficult to balance different responsibilities because of my child(ren)
13	The behaviour of my child(ren) is often embarrassing or stressful to me
14	If I had it to do over again, I might decide not to have child(ren)
15	I feel overwhelmed by the responsibility of being a parent
16	Having child(ren) has meant having too few choices and too little control over my life
17	I am satisfied as a parent
18	I find my child(ren) enjoyable

The following statements describe feelings and perceptions about the experience of being a parent. Think of each of the items in terms of how your relationship with your child or children typically is. Please indicate the degree to which you agree or disagree with the following items by placing the appropriate number in the space provided.

1 = Strongly disagree 2 = Disagree 3 = Undecided 4 = Agree 5 = Strongly agree.

### Sociodemographic predictor measures

The predictors were the following sociodemographic characteristics: income, education level, marital status, mother’s age, child’s age, and number of children aged < 5 years. Information on these were collected via a structured questionnaire. Mothers’ incomes are reported in bands (below USD 50, between USD 50 and 100, and above USD 100 per month). Mothers’ level of education was categorised as primary, secondary, and postsecondary education, which included college and vocational education. Marital status was categorised as married (living together/cohabiting, legally married) or unmarried (single, divorced, separated, or widowed). The number of children aged < 5 years under care included all those living in the same household, including nonbiological children.

### Statistical analysis

Data cleaning and analysis were performed using the R software and R Studio^46^. A multiple linear regression model was used to determine the association between mothers’ parenting stress scores and their sociodemographic characteristics, with adjustments for clustering and the study arm (control/intervention) at each study site. In the unadjusted model, the outcome variable was PSS, while the predictor variables were income, education level, marital status, mother’s age, child’s age, and number of children aged < 5 years. In the adjusted model, we added covariates (clusters and study arms). The full analysis code is available at the Open Science Framework (https://osf.io/yfxjc).

### Ethics approval and consent to participate

Permission to use these datasets was granted by the African Population and Health Research Centre (APHRC). The APHRC obtained ethical approval from the Institutional Review Boards (IRBs) of Kenya and Zambia to conduct these studies at the three study sites. Written informed consent was obtained from the study participants before data were collected. For respondents who could neither read nor write, a thumbprint was used as a signature in the presence of a witness. Consent was obtained at every round of data collection. Consent documents and the questionnaire were translated into Kiswahili (Kenya urban study site), Dholuo (Kenya rural study site), and Nyanja and Tonga (Zambia rural study site). Confidentiality of the data and the participants’ privacy were always observed during and after data collection. These rural and urban studies were registered under the trial registration numbers PACTR20180774832663 and PACTR201905787868050, respectively. All methods were performed in accordance with the relevant guidelines and regulations of the Declaration of Helsinki. For example, respect for individuals, the right to make informed decisions, and the recognition of vulnerable groups.

### Informed consent process

The data collectors sought informed consent from all study participants before they were interviewed. For those who were not able to read, the information sheet was read to them in their local language, and they were asked to provide a thumbprint to signify their consent. Ethical research committees in both countries approved the use of a thumbprint or signature. The ethical research committees in both countries approved the use of a thumbprint or signature (Amref Health Africa’s Ethics and Scientific Review Committee in Kenya and the ERES Converge in Zambia).

### Patient and public involvement

To design and implement this study, national and regional stakeholders from the Ministry of Health and Education were involved as part of the study team. Data collectors were also recruited from the study community. Stakeholders participated in the selection of the study site based on health indicators.

## Results

### Descriptive statistics

Descriptive statistics for sociodemographic characteristics are provided in Table 2. Mothers had a mean age of approximately 27 years in the two rural populations, whereas the urban population had a slightly higher mean age of approximately 29 years. More than 70% of the mothers reported that they had a marital relationship. Notably, most mothers reported that their educational level was secondary school or lower. Regarding their monthly incomes, most participants from the urban population reported a higher income (above USD 100) than the participants in the rural study sites. Slightly more than 60% of the participants reported that they had one child less than 5 years of age.

**Table 2:** Descriptive statistics on sociodemographic characteristics and mean PSS.

	Rural Kenyan	PSS Mean (SD)	Rural Zambian	PSS Mean (SD)	Urban Kenyan	PSS Mean (SD)
	n = 231 (%)		n = 378 (%)		n = 235 (%)	
Income						
Below USD 50	98 (42.4)	33.9 (12.2)	45 (11.9)	40.1 (8.09)	11 (4.7)	50.4 (4.1)
USD 50–100	133(57.6)	37.3 (10.0)	327 (86.5)	43.3 (9.38)	166(70.6)	48.4 (3.9)
Above USD 100	0 (0)		6 (1.6)	41.3 (7.30)	58(24.7)	47.4 (4.7)
Education						
Primary and below	80 (34.6)	40.9 (10.3)	226(59.8)	42.7 (9.21)	147 (62.6)	48.8 (4.1)
Secondary	143 (61.9)	35.8 (10.3)	152(40.2)	43.6 (9.05)	77 (32.8)	47.6 (4.5)
Above secondary	8 (3.5)	38.5 (10.8)			11 (4.7)	46.8 (4.2)
Marital status						
Single parent	28 (12.1)	44.8 (11.0)	100(26.5)	43.8 (8.43)	25 (10.6)	47.7 (4.7)
Married	203 (87.9)	36.4 (11.8)	278(73.5)	42.5 (9.35)	210 (89.4)	48.4 (4.1)
Mother age						
Below 20 years	32 (13.9)	38.2 (12.6)	49(13.0)	43.2 (8.61)	16 (6.8)	48.9 (3.0)
20–29	117 (50.6)	35.8 (10.4)	205(54.2)	42.0 (8.56)	119 (50.6)	47.7 (4.3)
30–35	68 (29.4)	39.6 (11.5)	97(25.6)	42.4 (9.60)	86 (36.6)	48.9 (4.2)
Above 35	14 (6.1)	48.2 (8.8)	27(7.1)	43.3 (11.2)	14 (6.0)	49.9 (4.1)
Child mean age in years (SD)	0.75 (0.7)	39.7 (10.8)	1.0 (0.6)	42.4 (9.05)	1.4 (0.4)	48.1 (4.12)
Number of children aged < 5						
1	156 (67.5)	37.7 (11.6)	240 (63.5)	42.0 (8.85)	230 (97.9)	48.5 (4.2)
2	53 (22.9)	37.3 (10.2)	76 (20.1)	43.8 (9.92)	2 (0.9)	49.6 (3.3)
3 and 4	8 (3.5)	41.0 (13.1)	62 (14.4)	47.0 (9.83)	3 (1.3)	48.0 (5.7)

**Figure 2:**
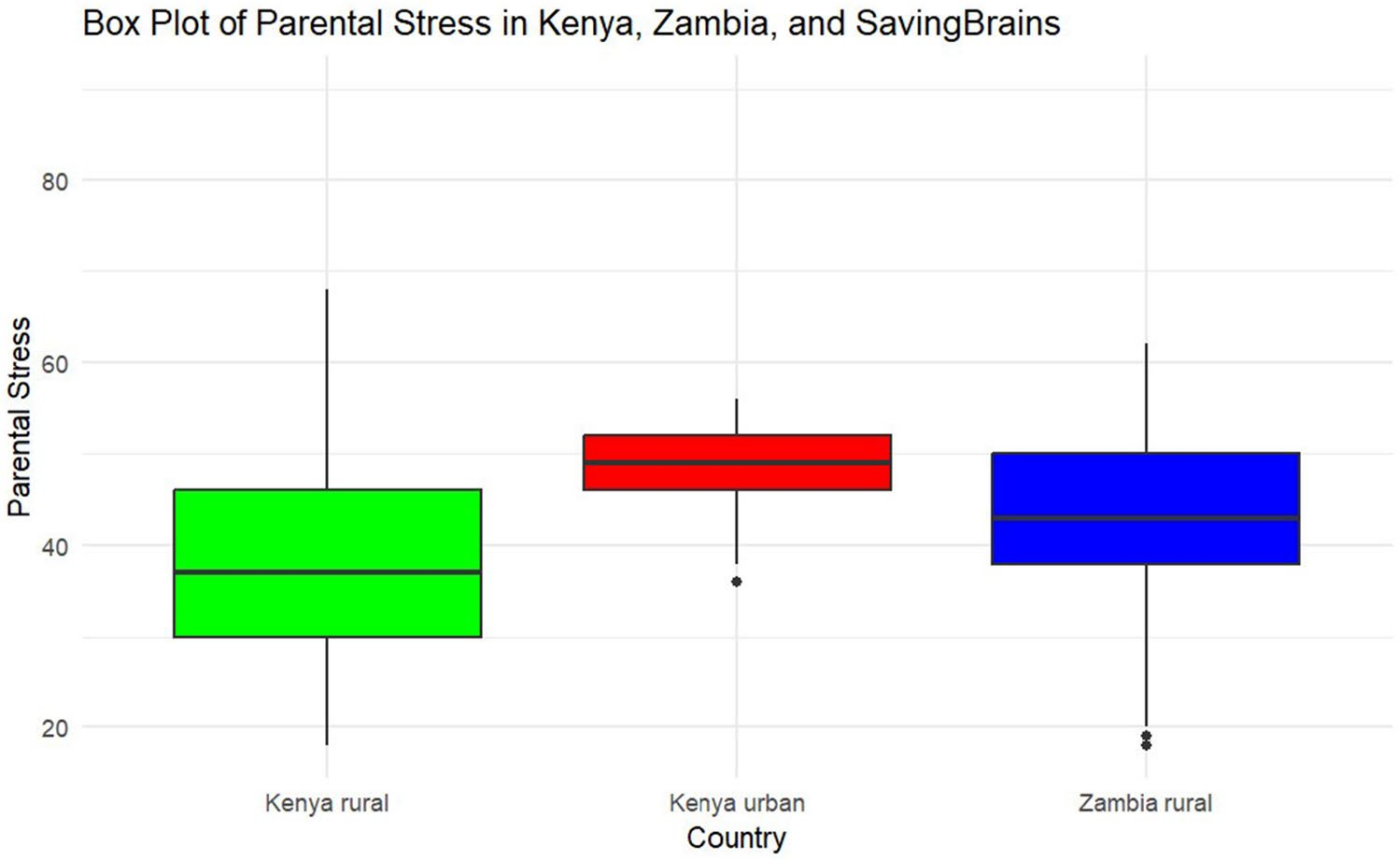
Mean parental stress score.

The mean PSS were higher at Kenya’s urban study site than at the two rural study sites (Kenya rural: 37.6 [SD = 11.8], Kenya urban: 48.4 [SD = 4.2], and Zambia rural: 43.0 [SD = 9.1]), as shown in Fig. 2. Notably, in the Kenya rural study site, children’s mean age (in years) at preintervention was 0.8 (SD = 0.7) in the Kenyan rural site, while in the Zambia rural study site, children’s mean age at preintervention was 0.9 (SD = 0.6) in the Zambia site and 3.4 (SD = 0.4) in the urban Kenyan population.

### Modelling results on sociodemographic predictors of PSS

We also sought to establish associations between household income, educational level, marital status, maternal age, child age, number of children under five years of age, and mothers’ PSS.

The results of the unadjusted linear regressions showed that in Kenya’s rural study site, the mean PSS of mothers with an income above USD 50 was 0.428 standard deviations lower than that of mothers with a monthly income of less than USD 50 (β = − 0.428 [95% CI − 1.483, − 0.518], *p* < 0.01***). A similar trend was observed at Kenya’s urban study site, with a standard deviation of 0.341 (β = − 0.341 [95% CI − 1.638, − 0.221], *p* = 0.01*).

When adjusted for clustering and study arm, the results from Kenyan rural sites showed that the mean PSS of mothers with income above USD 50 was 0.403 standard deviation lower than that of mothers with a monthly income less than USD 50 (β = − 0.403 [95% CI − 1.422, − 0.455], *p* < 0.01***). A similar trend was observed in Kenya's urban study site, with a standard deviation of 0.325 (β = − 0.325 [95% CI − 1.598, − 0.174], *p* = 0.02*), as shown in Tables 3, 4, and 5. Notably, the income category ‘above USD 100’ was excluded from Kenyan rural study site modelling, as no participants were in this category. Similarly, the above secondary education was excluded from the Zambia rural study site model for the same reason.

**Table 3:** Beta (B), standard errors (SE), probability values (P) and standardised betas (β) for unadjusted and adjusted models on associations between prenatal demographic characteristics and parental stress Kenya rural study site.

Variable	Level	Unadjusted			Adjusted		
		β	[95% CI]	*p* value	β	[95% CI]	*p* value
Income	Below USD 50	Reference					
	USD 50–100	− 0.428	[− 1.483, − 0.518]	< 0.001***	− 0.403	[− 1.422, − 0.455]	< 0.001***
Education	Primary and below	Reference					
	Secondary	− 0.262	[− 0.866, − 0.187]	0.01**	− 0.250	[− 0.838, − 0.667]	0.01**
	Above secondary	− 0.053	[− 1.122, 0.560]	0.5	− 0.014	[− 0.922, 0.775]	0.5
Marital status	Single	Reference					
	Married	− 0.037	[− 0.555. 0.380]	0.7	− 0.043	[− 0.562, 0.357]	0.7
Mother age	Below 20 years	Reference					
	20–29	0.151	[− 0.241, 0.842]	0.3	0.136	[− 0.272, 0.813]	0.2
	30–35	0.196	[− 0.155, 1.001]	0.1	0.183	[− 0.177, 0.967]	0.1
	Above 35	0.064	[− 0.492, 0.980]	0.5	0.063	[− 0.483, 0.963]	0.5
Child age		− 0.017	[− 0.172, 0.138]	0.8	− 0.012	[− 0.166, 0.141]	0.8
Number of children aged < 5	1	Reference					
	2	− 0.022	[− 0.418, 0.320]	0.8	− 0.009	[− 0.384, 0.342]	0.9
	3	0.077	[− 0.382, 1.123]	0.3	0.086	[− 0.329, 1.155]	0.2

*Significant at *P* > .05. All the adjusted models were controlled for clustering. The Parental Stress Score tool ranges from 18 to 90, with a low score indicating a low parental stress level. Therefore, the B coefficients should be interpreted accordingly. Standardised betas (β) were additionally provided to allow for direct comparisons of effect sizes. *Significant at *P* < 0.05, **Significant at *P* < 0.01 and ***Significant at *P* < 0.001.

**Table 4:** Beta (B), standard errors (SE), probability values (P) and standardised betas (β) for unadjusted and adjusted models on associations between prenatal demographic characteristics and parental stress Kenya urban study site.

Variable	Level	Unadjusted			Adjusted		
		β	[95% CI]	*p* value	β	[95% CI]	*p* value
Income	Below USD 50	Reference					
	USD 50–100	− 0.254	[− 1.237, 0.006]	0.05*	− 0.234	[− 1.197, 0.062]	0.07
	Above USD 100	− 0.341	[− 1.638, − 0.221]	0.01**	− 0.325	[− 1.598, − 0.174]	0.02*
Education	Primary and below	Reference					
	Secondary	− 0.147	[− 0.622, 0.014]	0.06.	− 0.140	[− 0.607, 0.030]	0.07.
	Above secondary	− 0.021	[− 0.816, 0.619]	0.8	− 0.006	[− 0.753, 0.693]	0.8
Marital status	Single	Reference					
	Married	0.019	[− 0.386, 0.497]	0.8	0.007	[− 0.427, 0.469]	0.8
Mother age	Below 20 years	Reference					
	20-29	− 0.157	[− 0.920, 0.294]	0.3	− 0.157	[− 0.929, 0.301]	0.3
	30-35	− 0.005	[− 0.608, 0.629]	0.9	− 0.005	[− 0.624, 0.646]	0.9
	Above 35	0.064	[− 1.105, 0.560]	0.5	− 0.058	[− 1.078, 0.587]	0.5
Child age		0.018	[− 0.127, 0.163]	0.8	0.025	[− 0.120, 0.170]	0.8
Number of children aged <5	1	Reference					
	2	0.065	[− 1.772, 3.042]	0.8	0.050	[− 1.726, 3.085]	0.8
	3	− 0.067	[− 2.676, 1.725]	0.6	− 0.070	[− 2.721, 1.695]	0.6

All the adjusted models were controlled for clustering. The Parental Stress Score tool ranges from 18 to 90, with a low score indicating a low parental stress level. Therefore, the B coefficients should be interpreted accordingly. Standardised betas (β) were additionally provided to allow for direct comparisons of effect sizes. *Significant at *P* < 0.05, **Significant at *P* < 0.01 and ***Significant at *P* < 0.001.

**Table 5:** Beta (B), standard errors (SE), probability values (P) and standardised betas (β) for unadjusted and adjusted models on associations between prenatal demographic characteristics and parental stress Zambia rural study site.

Variable	Level	Unadjusted			Adjusted		
		β	[95% CI]	*p* value	β	[95% CI]	*p* value
Income	Below USD 50	Reference					
	USD 50–100	0.097	[− 0.120, 0.755]	0.2	0.099	[− 0.100, 0.760]	0.2
	Above USD 100	0.028	[− 1.225, 0.801]	0.7	− 0.010	[− 1.092, 0.937]	0.7
Education	Primary and below	Reference					
	Secondary	0.063	[− 0.122, 0.378]	0.3	− 0.042	[− 0.167, 0.337]	0.3
Marital status	Single	Reference					
	Married	0.384	[− 0.364, 0.190]	0.5	− 0.033	[− 0.350, 0.200]	0.6
Mother age	Below 20 years	Reference					
	20–29	− 0.098	[− 0.573, 0.181]	0.3	− 0.095	[− 0.565, 0.184]	0.3
	30–35	− 0.065	[− 0.554, 0.264]	0.5	− 0.060	[− 0.541, 0.274]	0.5
	Above 35	− 0.023	[− 0.637, 0.463]	0.8	− 0.030	[− 0.658, 0.436]	0.8
Child age		0.093	[− 0.028, 0.213]	0.1	0.075	[− 0.046, 0.195]	0.2
Number of children aged < 5	1	Reference					
	2	− 0.117	[− 0.016, 0.653]	0.06	0.106	[− 0.045, 0.622]	0.09
	3	− 0.061	[− 0.491, 1.534]	0.3	0.065	[− 0.451, 1.563]	0.3

*Significant at *P* > 0.05. All the adjusted models were controlled for clustering. The Parental Stress Score tool ranges from 18 to 90, with a low score indicating a low parental stress level. Therefore, the B coefficients should be interpreted accordingly. Standardised betas (β) were additionally provided to allow for direct comparisons of effect sizes. *Significant at *P* < 0.05, **Significant at *P* < 0.01 and ***Significant at *P* < 0.001.

The results from the unadjusted linear regressions showed that, in Kenya’s rural study site, the mean PSS of mothers with secondary education was 0.26 a standard deviation lower than that of mothers with primary education (β = − 0.262 [95% CI − 0.866, − 0.187], *p* = 0.01**). The findings from Kenya’s urban and Zambia’s rural sites showed no association between education and parental stress (Kenya’s urban study site: β = − 0.147 [95% CI − 0.622, 0.014], *p* = 0.06; Zambia rural: β = − 0.63 [95% CI − 0.122, 0.378], *p* = 0.3).

When adjusted for clustering and study arm, the results from Kenyan rural sites showed that the mean PSS of mothers with secondary education was 0.25 a standard deviation lower than that of mothers with primary education (β = − 0.250 [95% CI − 0.838, − 0.667], *p* = 0.01**). The findings from Kenya’s urban and Zambia’s rural sites showed no association between education and parental stress (Kenya’s urban study site: β = − 0.14 [95% CI − 0.607, 0.030], *p* = 0.07; Zambia rural: β = − 0.042 [95% CI − 0.167, 0.337], *p* = 0.3). In addition, the findings on other predictors, such as marital status, mother’s age, child age, and number of children aged < 5 years, showed no significant association with PSS across the study sites as shown in Tables 3, 4, and 5.

## Discussion

This study aimed to establish predictors of parental stress using baseline datasets from three studies in two African countries (rural Kenya, rural Zambia, and urban Kenya). The results showed that parental stress was associated with at least two factors: the mother’s income and educational level. In addition, the mean PSS were slightly above scores observed in a study in a low-resourced setting in South Africa in which the baseline mean PSS for the treatment group was 33.1 (SD = 8.7) and the control group was 33.4 (SD = 8.2). We speculate that the higher PSS in Kenya and Zambia settings could be attributed to a higher proportion of the population living in low socioeconomic status compared to South Africa. In addition, cultural variations within communities could have influenced parenting practices and, thereby, variation in PSS.

Findings on high PSS, especially in the poor urban population, as observed in this study, could illuminate the challenges encountered by women in combining childcare and work in poor urban settings, as reported in other studies^47^. Such scores were not reported in rural Zambia and Kenya because of communal childcare practices. That is, the childcare burden is often spread among extended family members, such as grandparents and other relatives^48^. Mothers can leave their children to their grandparents or other relatives while working and carrying out household chores. However, such practices are uncommon in urban settings, where mothers often take their children to childcare facilities when they go to work. Notably, the quality of such childcare services is often poor, which could cause parents to worry about whether their child is receiving the right services^49^. Therefore, we speculate that this could be the reason for the higher parental stress at the Kenyan urban study site.

Our findings on the predictors of PSS established that the PSS was associated with the mothers’ income in Kenyan study sites. These findings extend those of other studies that have shown associations between household income and mothers’ PSS^50^. Such findings were also observed in a study conducted on perceived stress related to COVID-19, in which mothers from low-income households reported more uncertainty about their health than their counterparts with higher incomes^51^. Notably, these studies have been carried out in other contexts that do not represent sub-Saharan Africa. Although the findings of the current study were not consistent across the study sites, it is notable that the mean difference in PSS was consistent across the study sites. Therefore, these findings highlight the need for maternal health and childcare interventions to reduce parental stress levels by improving household incomes and reducing childcare-related expenditures, such as providing quality and affordable childcare services.

An association between PSS and mothers’ education levels were also observed, though varied across the study sites. These results were similar to another study of sociodemographic predictors of chronic stress in mothers^22^. To enhance parenting skills and reduce stress, it is suggested that mothers with less formal education could benefit from engaging in parenting programs, which have been shown to positively impact their children’s development^52^. Therefore, community parenting programs or social groups could assist parents in sharing their parenting challenges and coming up with potential solutions, thereby reducing the stressors that come with parenting. Surprisingly, the number of children aged < 5 years under care was not associated with parental stress at the three study sites. However, similar findings on parental burnout in SSA have been reported in a study conducted in Somalia among women seeking Maternal and Child Health (MCH) services^53^ and in Uganda among parents of primary school children^54^. In addition, the findings on marital status, mother's age, and the age of the youngest child were also surprising. Notably, we could not find any study on sociodemographic predictors of parental stress in the SSA context for comparison with our findings. Therefore, future studies could generate further evidence on the specific sociodemographic predictors of PSS among parents in socially deprived settings.

### Study limitations

Despite using three study sites that present diverse, disadvantaged populations in SSA, we could not draw a conclusive link between parental stress and mothers’ sociodemographic characteristics. Noting that the population represented in this study was from three distinct studies with different study designs and geographical locations, it might be difficult to separate naturally occurring effects from regional and study-design effects. Another limitation was that the mothers’ sociodemographic characteristics were based on self-reporting, which could have introduced reporting biases. In addition, our study focused on mothers’ parenting stress. Considering the potential contributions of paternal parenting stress to parenting practices and children’s developmental outcomes, future studies could focus on investigating predictors of paternal parenting stress and its effects on parenting practices and children’s developmental outcomes. Therefore, these findings are indicative of the association between mothers’ socio-demographic characteristics and PSS but do not prove causality.

## Conclusions and policy implications

This study aimed to determine the predictors of parental stress among mothers in low-resource SSA settings. The findings indicated that parental stress was associated with at least two factors: the mother’s income and educational level. However, PSS was not related to other sociodemographic factors such as marital relationship, age of the child, age of the mother, and number of children aged below five years under their care. If our findings are replicated in the same setting, it will be prudent for interventions to focus on improving maternal mental health through poverty-alleviation-related interventions, such as subsidising childcare services and improving household income in poor urban and rural settings.

## Data Availability

The datasets used and analysed during the current study are available from the corresponding author upon reasonable request.
